# Morphological variability of the plantaris tendon in the human fetus

**DOI:** 10.1038/s41598-021-96391-8

**Published:** 2021-08-19

**Authors:** Anna Waśniewska-Włodarczyk, Friedrich Paulsen, Łukasz Olewnik, Michał Polguj

**Affiliations:** 1 Department of Normal and Clinical Anatomy, Chair of Anatomy and Histology, Medical University of Lodz, Lodz, Poland; 2 Institute of Functional and Clinical Anatomy, Friedrich Alexander University Erlangen-Nürnberg, Erlangen, Germany; 3 Department of Topographic Anatomy and Operative Surgery, Sechenov University, Moscow, Russia; 4 Department of Anatomical Dissection and Donation, Medical University of Lodz, Lodz, Poland

**Keywords:** Anatomy, Evolutionary developmental biology

## Abstract

Muscular anatomy often differs between species and individuals. In particular, the plantaris muscle (PM) demonstrates great morphological variability in its course and its proximal and distal attachments. The aim of this study was to investigate the morphological variation of the PM tendon in human fetuses. Forty-six spontaneously aborted human fetuses (23 male, 23 female) aged 18–38 weeks of gestation were studied. Morphology of the attachment of the PM was assessed in both lower extremities (n = 92). The PM was present in 72 lower limbs (78.26%) and absent in 20 (21.74%). Eight types of PM distal attachment were identified. We propose an eight-fold classification of PM insertion in fetuses. Leg length, length of tendon, extension point (ExP) from the calcaneus, and ExP thickness differed significantly among types of PM insertion.

## Introduction

Muscular variations and anomalies in humans with congenital malformations have been frequently analyzed since the nineteenth century. Several muscles thought to have been lost in our adult ancestors are often observed in early ontogenetic stages. In most people, however, they are reabsorbed or fused with other structures before birth^1^. The structure of muscles often varies between species and within them^2^. In particular, the plantaris muscle (PM) demonstrates great morphological variability in its course and proximal and distal attachments^3,4^.

While there are several classifications of insertion type, these may not cover certain extremely rare examples of PM, and accurate identification is crucial for correct differentiation and diagnosis of certain conditions, as well as for patient health^5–8^. The tendon of the PM is often used in reconstruction of the tendons and stabilization of the joints. This muscle has also influence on the midportion Achilles tendinopathy^9–11^. There is possibility of rupture of PM’s belly and tendon at the muscular-tendon junction, which may be classified as tennis leg, a term that may also describe rupture of the GM^9–11^. Moreover, release of PM tendon should be considered in all types equinus^12,13^. Therefore, getting familiar with morphology of the PM is so important.

Despite its small size and negligible impact on movement, as well as the possibility that it is indeed a vestigial muscle^10,14,15^, the PM nevertheless exhibits high clinical significance. However, most studies on the morphology and morphometry of the PM have been performed in adults, and data on its structure in human fetuses are sparse. Previous studies were performed on fetuses by Yildiza et al.^16^ and Desdicioglu et al.^17^, nevertheless neither Yildiza et al. nor Desdicioglu et al. identified all insertion types of PM tendon, these studies were focused on measurements of PM tendon, not the classification and comparison with adults. Therefore, the aim of this study was to assess the morphology of the tendon of the PM in human fetuses and to compare the results with those of other studies performed in fetuses and adult cadavers. Knowing the morphology and morphometry of PM tendon in human fetuses will be beneficial for better understanding the biomechanics of this muscle.

## Materials and methods

Forty-six spontaneously aborted human fetuses (23 female, 23 male) between 18 and 38 weeks of gestation were studied. The fetuses were obtained after parental informed consent was obtained and were from the Department of Anatomical Dissection and Donation Collection. The study was conducted in accordance with the cadaveric donation program for both adults and fetuses and with the legal procedures in force in Poland. The age of the fetuses was determined from the head and craniosacral measurements. The study was approved by the local bioethics committee at the Medical University of Łódź (agreement no. RNN/218/20/KE).

The leg and foot were dissected using traditional techniques^18–20^. The subcutaneous tissue was removed to expose the gastrocnemius muscle (GM) and separate it from the soleus muscle (SM). The structures around the PM were then cleaned.

The following characteristics were recorded:

1. Length of the leg—maximal length measured from the top of medial condyle of the tibia to the end of the medial malleolus.
2. Type of the PM tendon insertion.
3. Length of the PM tendon—maximal length measured from the junction of the muscle and the tendon to the attachment of the PM tendon.
4. The thickness and width of the PM at the extension point (ExP) and the distance between this point and the attachment of the PM tendon. The ExP is the point at which the distal tendon begins to expand before its insertion; its presence depends on the type of insertion of the tendon.

Measurements were made with an electronic digital caliper (Mitutoyo Corporation, Kawasaki-shi, Kanagawa, Japan). Each measurement was performed twice with an accuracy of up to 0.01 mm.

### Statistical analysis

The collected tendon measurements were compared using the Statistica 13.1 software package (StatSoft, Cracow, Poland). Analysis of variance (one-way ANOVA or Kruskal–Wallis test) followed by post-hoc tests (HSD Tukey) was used to calculate differences. Shapiro–Wilk’s *W* test was used to test the distribution of variables. Comparisons between groups were performed with Student’s t-test (or nonparametric Mann–Whitney *U*-test) and chi2 test. A value of P < 0.05 was considered statistically significant.

### Classification

The classification of the insertion of the PM tendon was based on the classification of Olewnik et al.^4,11^. Based on this classification, we distinguished six same types, and two types absent in that study.

**Figure 1:**
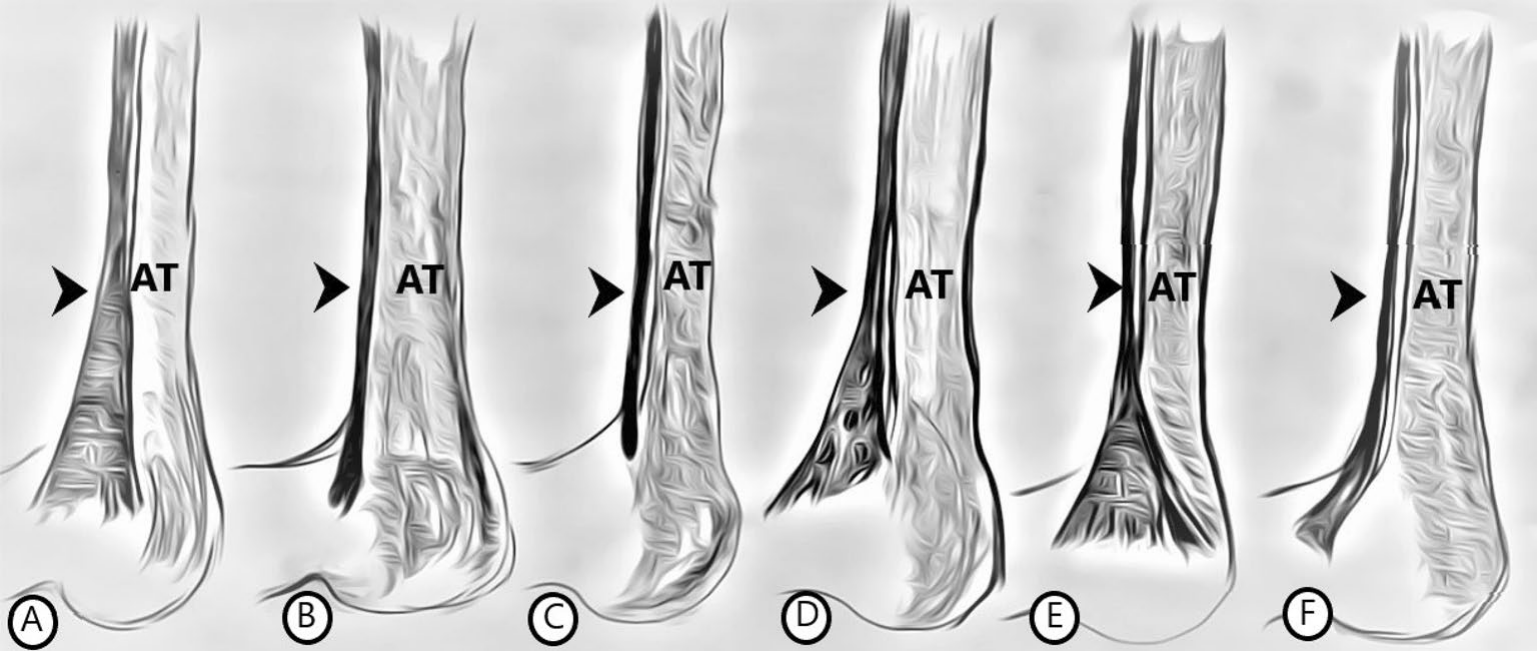
Types of tendon attachments. (**A**) type I, (**B**) type II, (**C**) type III, (**D**) type IV, (**E**) type V, (**F**) type VI. *AT *Achilles tendon,* Arrow* plantaris tendon.

**Figure 2:**
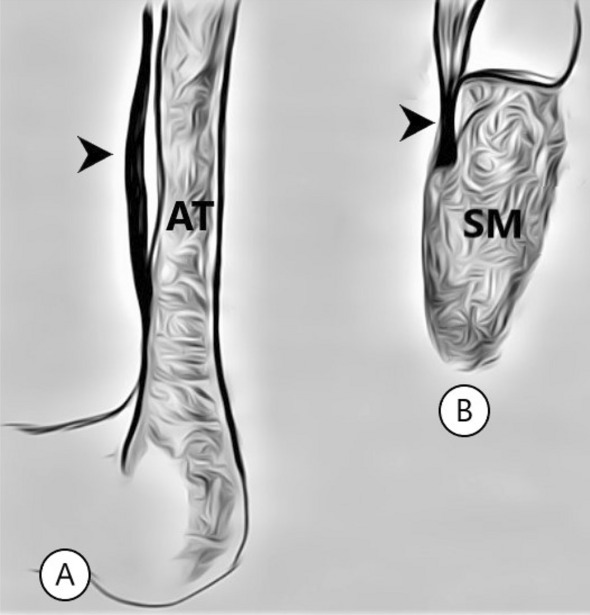
Types of tendon attachments. (**A**) type VII, (**B**) type VIII. *AT *Achilles tendon, *Arrow* plantaris tendon, *SM* soleus muscle.

1. Type I—the tendon is fan-shaped. It inserts medially to the Achilles tendon, but separately from it, on the calcaneal tuberosity. Fig. 1A.
2. Type II—the tendon is band-shaped. It inserts medially on the Achilles tendon and adheres closely to it in a single paratendon. Fig. 1B.
3. Type III—the tendon inserts anterior to the Achilles tendon. Fig. 1C.
4. Type IV—the tendon inserts at the deep crural fascia. Fig. 1D.
5. Type V—the insertion is broad and overlies the Achilles tendon. Fig. 1E.
6. Type VI—the tendon attaches to the flexor retinaculum of the leg. Fig. 1F.
7. Type VII—the tendon inserts at the Achilles tendon. Missing in the studies by Olewnik et al.^4,11^. Fig. 2A.
8. Type VIII—the tendon is short and attaches to the SM. Missing in the studies by Olewnik et al.^4,11^. Fig. 2B.

## Results

### Frequency of occurrence of the PM

The PM was present in 72 lower limbs (78.26%) and absent in 20 limbs (21.74%). It was also found to be absent in eleven males and nine females (ten left limbs and ten right limbs). No significant difference was found in the frequency of absence between sex (p = 0.613) or between body sides (p = 1). No significant difference was found in the type of PM tendon between sex (p = 0.053) or between body sides (p = 0.48). The relationship between specific type of insertion and sex and body side is shown in more detail in Table 1.

**Table 1:** Occurrence of all eight identified types with relation to sex and body side.

Type	Sex		Body side	
	Male (n = 35)	Female (n = 37)	Right	Left
I	4	5	5	4
	11.43%	13.51%	13.89%	11.11%
II	11	9	9	11
	31.43%	24.32%	25.00%	30.56%
III	1	1	0	2
	2.86%	2.70%	0.00%	5.56%
IV	10	9	12	7
	28.57%	24.32%	33.33%	19.44%
V	0	8	3	5
	0.00%	21.62%	8.33%	13.89%
VI	0	2	0	2
	0.00%	5.41%	0.00%	5.56%
VII	7	3	6	4
	20.00%	8.11%	16.67%	11.11%
VIII	2	0	1	1
	5.71%	0.00%	2.78%	2.78%
p-value	0.053		0.480	

### Course of the PM tendon

The PM tendon crossed the space between the GM and SM in all types, with one exception: in this type, the tendon was extremely short and ended in the SM.

### Evaluation of the tendon of the PM

Eight key types were distinguished based on PM morphology. Six types were based on the classification of Olewnik et al.^4,11^ and two types were absent in those studies.

**Figure 3:**
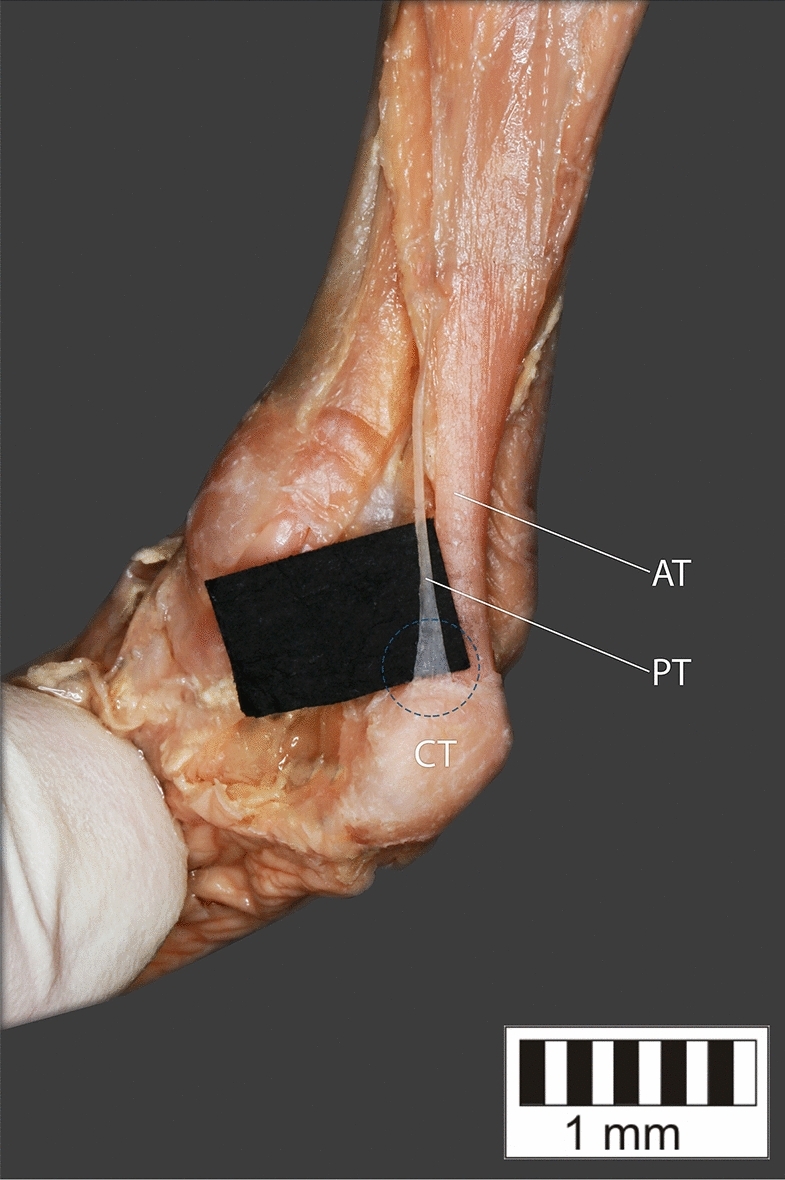
Type I of plantaris tendon. *CT *calcaneal tuberostity,* AT *Achilles tendon,* PT *plantaris tendon.

**Figure 4:**
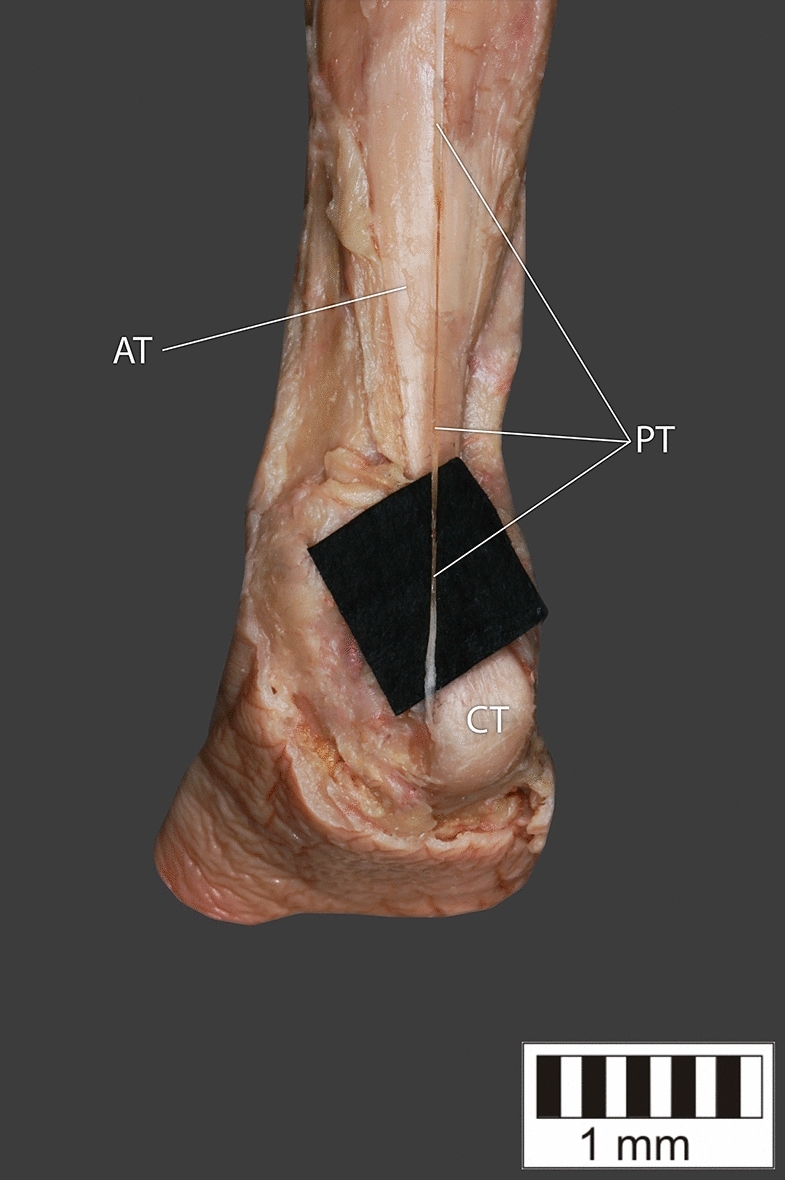
Type II of plantaris tendon. *CT* calcaneal tuberostity, *AT* Achilles tendon, *PT* plantaris tendon.

**Figure 5:**
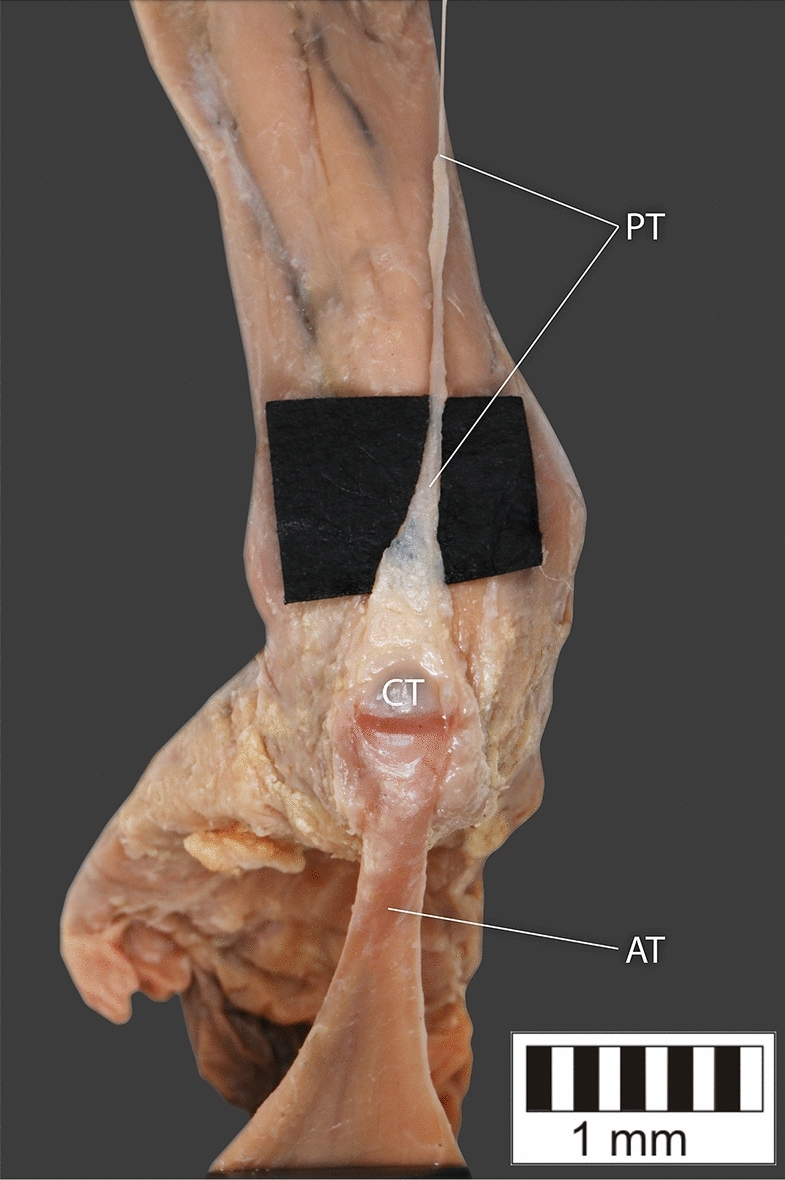
Type III of plantaris tendon. *CT* calcaneal tuberostity, *AT* Achilles tendon, *PT* plantaris tendon.

**Figure 6:**
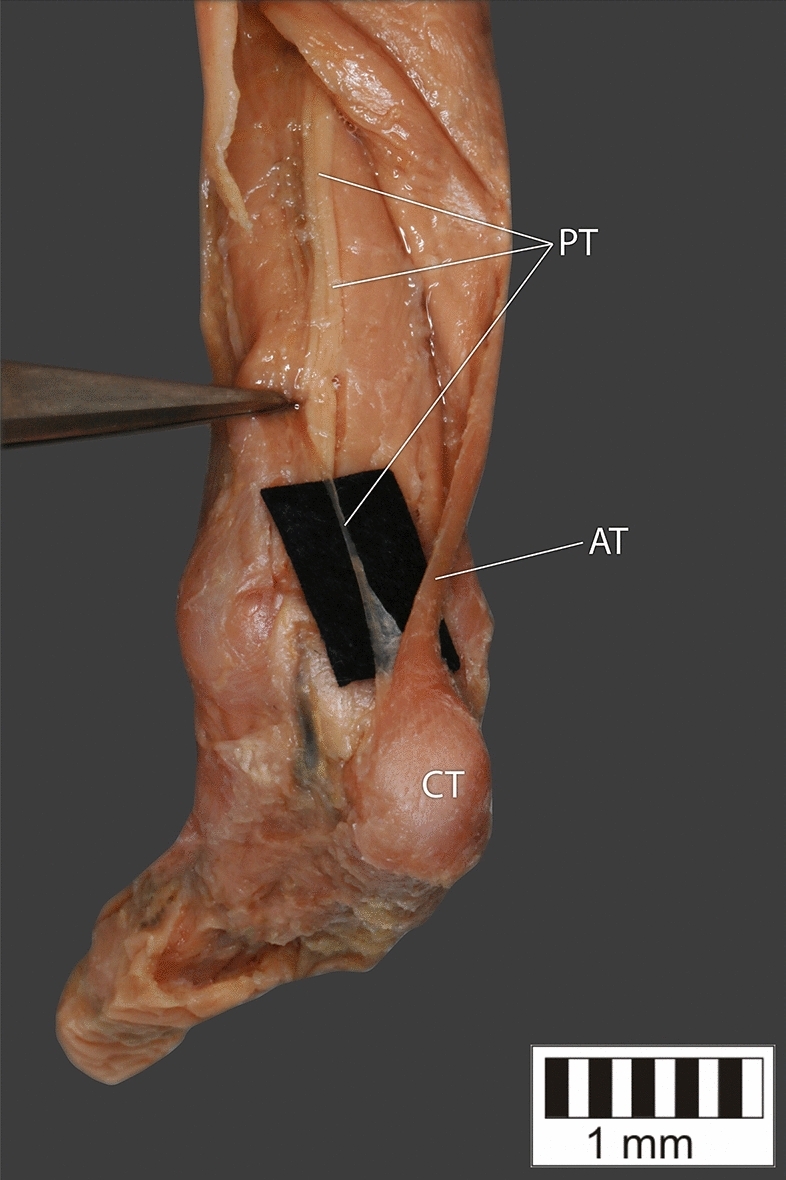
Type IV of plantaris tendon. *CT* calcaneal tuberostity, *AT* Achilles tendon, *PT* plantaris tendon.

**Figure 7:**
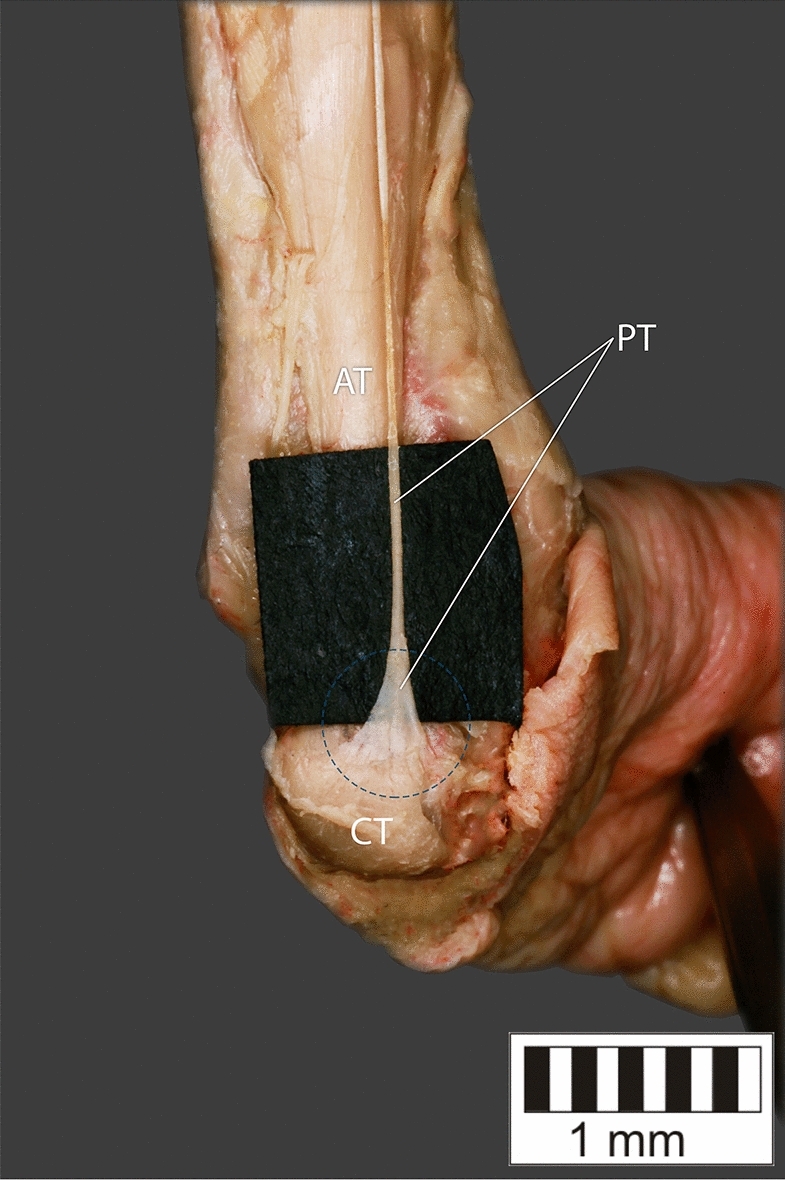
Type V of plantaris tendon. *CT*- calcaneal tuberostity, *AT* Achilles tendon, *PT* plantaris tendon.

**Figure 8:**
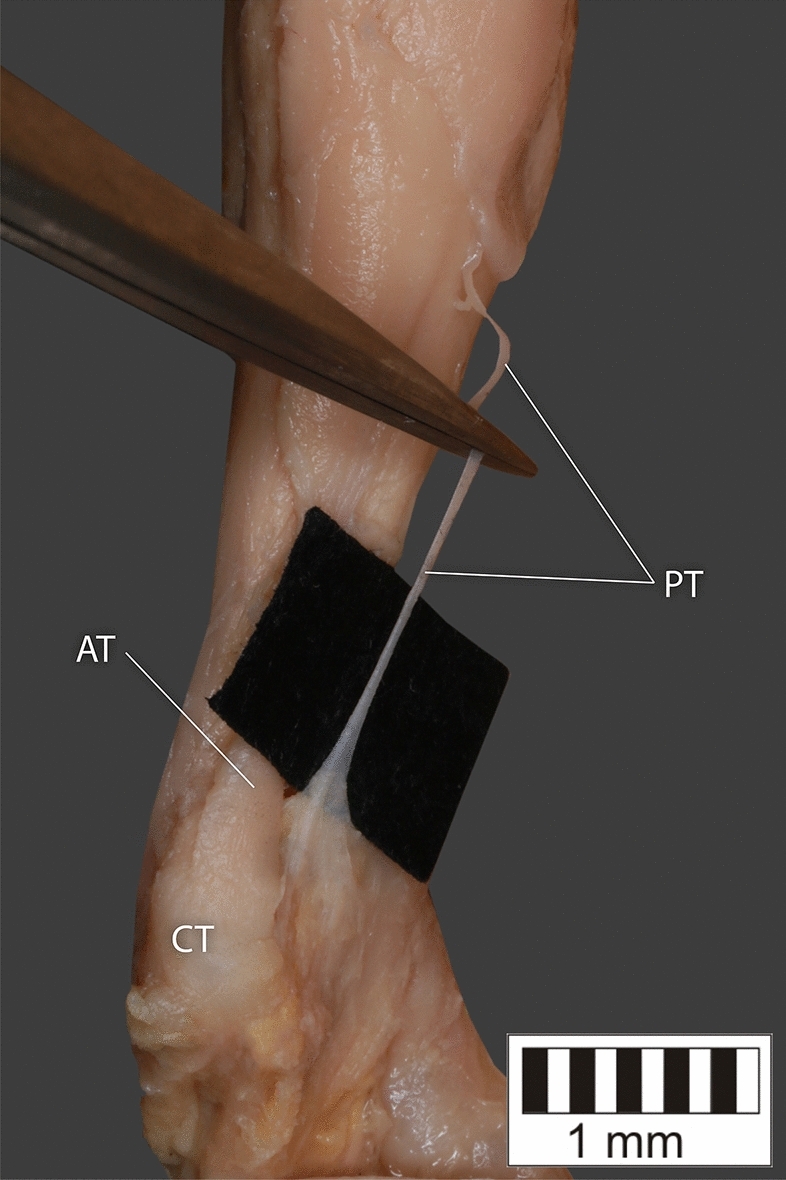
Type VI of plantaris tendon. *CT* calcaneal tuberostity, *AT* Achilles tendon, *PT* plantaris tendon.

**Figure 9:**
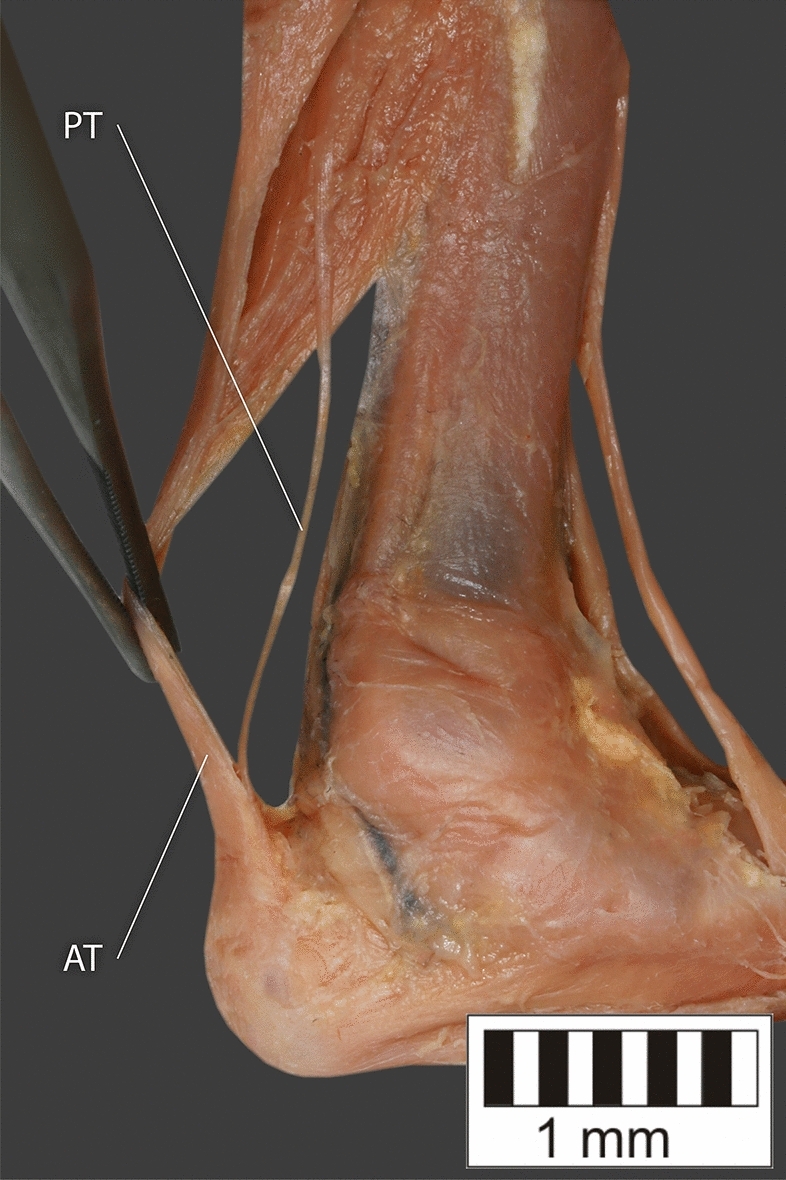
Type VII of plantaris tendon. *CT* calcaneal tuberostity, *AT* Achilles tendon, *PT* plantaris tendon.

**Figure 10:**
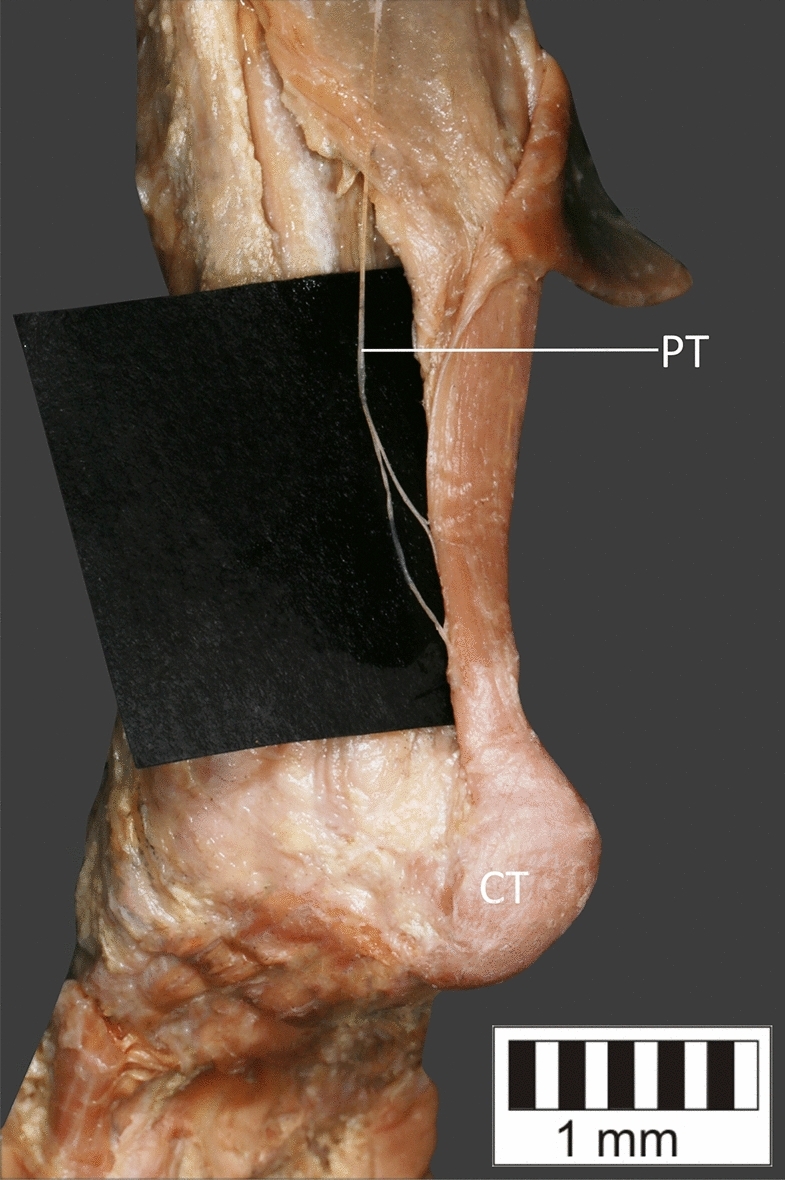
Type VII of plantaris tendon. PM ended with two bands both of which terminated in the Achilles tendon.

**Figure 11:**
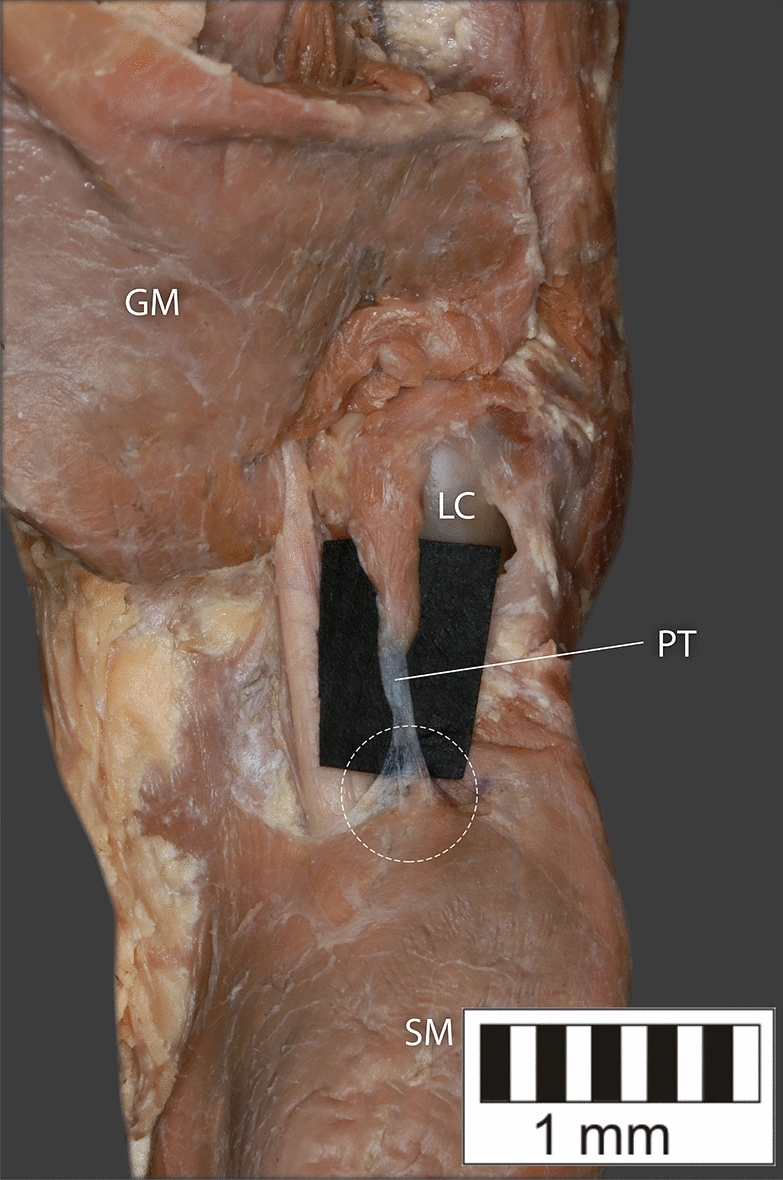
Type VIII of plantaris tendon. *SM* soleus muscle, *GM* gastrocnemius muscle, *PT* plantaris tendon, *LC* lateral condyle.

- Type I—Present on 9 lower limbs (9.78%)—Fig. 3.
- Type II—Present on 20 lower limbs (21.74%)—Fig. 4.
- Type III—The tendon attaches anterior to the Achilles tendon. Present on 2 lower limbs (2.17%)—Fig. 5.
- Type IV—Present on 19 lower limbs (20.65%)—Fig. 6.
- Type V—Present on 8 lower limbs (8.7%)—Fig. 7.
- Type VI—Present on 2 lower limbs (2.17%)—Fig. 8.
- Type VII—Present on 10 lower limbs (10.87%). The tendon inserts at the Achilles tendon. In one case, the PM tendon was divided into two ligaments before attachment to the Achilles tendon—Figs. 9, 10.
- Type VIII—Present on 2 lower limbs (2.17%). The tendon is short and attaches to the SM—Fig. 11.

The mean length of tendon of PM in the second trimester was 42.46 ± 6.94 mm, and the mean tendon length in third trimester was 54.29 ± 21.96 mm. According to one-way ANOVA leg length (F_7,64_ = 3.67, p = 0.002), tendon length (F_7,64_ = 5.04, p = 0.001), ExP from the calcaneus (F_6,24_ = 9.17, p < 0.001), and ExP thickness (F_6,26_ = 5.83, p < 0.001) differed significantly between types of PM insertion. As assessed by post hoc HSD Tukey test leg length was statistically the longest in type VIII (83.04 mm) compared to all other types. Type VIII was characterized by the shortest tendon (5.54 ± 1.4 mm) and was significantly differ than other types. The longest PT was observed in type VII (52.74 ± 11.34 mm). In addition, type VII had the longest distance between ExP to calcaneus (23.88 mm) and significantly differed from other groups. A post hoc Tukey test showed that thickness of ExP in type I (1.48 ± 0.6 mm) vs. IV (0.48 ± 0.48 mm), type IV (0.48 ± 0.48 mm) vs. VII (3.19 mm) and type V (0.53 ± 0.65 mm) vs. VII (3.19 mm) were statistically different. All morphometric measurements of the leg and PM are shown by insertion type in Table 2.

**Table 2:** Mean morphometric measurements by PM type with standard deviation (SD).

	I	II	III	IV	V	VI	VII	VIII	p-value
Length of the leg (mm)	42.43	46.44	45.26	46.15	46.99	44.15	54.75	83.04	0.002
SD	5.4	13.69	3.94	9.63	12.88	6.5	13.64	0.00	
Length of the tendon (mm)	42.22	45.19	45.94	45.49	44.04	44.39	52.74	5.54	0.001
SD	4.24	12.52	7.42	9.49	10.7	11.10	11.34	1.4	
ExP from the calcaneus (mm)	7.55	3.84	5.03	8.7	6.46	6.98	23.88		< 0.001
SD	2.45	0.00	0.00	2.2	2.59	0.00	0.00		
ExP width (mm)	0.15	0.90	0.07	0.77	0.53	0.12	0.13		0.247
SD	0.09	0.00	0.00	0.66	0.42	0.00	0.00		
ExP thickness (mm)	1.48	0.81	1.12	0.48	0.53	0.74	3.19		< 0.001
SD	0.6	0.00	0.00	0.48	0.65	0.00	0.00		

Statistical significance from the one-way ANOVA.

*ExP* extension point.

## Discussion

The present study has two key values: it presents the first systematic classification of PM insertion and highlights its variability in human fetuses by classifying it as band-shaped or fan-shaped. Our classification is based on Olewnik et al.^11^, although it includes type that has not been described in studies of adult cadavers.

The PM plays a much more important role in nonhuman primates, where it is used to grasp objects. However, as a result of the evolution of a bipetal stance, the human foot became adapted to walking long distances^21,22^, and this change may have resulted in the PM becoming a vestigial muscle. During evolution, the PM has been classified as a muscle that became partially or completely nonfunctional^15,23^. Daseler and Anson propose that the PM was attached to the plantar aponeurosis in remote ancestors; however, over time, the calcaneum sank to the ground, providing more stability in a bipedal stance. This process may have caused the variation in insertion of the PM tendon observed today^15,24^. Our present results show the presence of an extremely short tendon ending in the SM in 2.17% of cases. The loss of the PM tendon may confirm that the PM is a rudimentary muscle.

Two theories have been proposed regarding the embryological development of the PM. The first states that the group of flexors begins to differentiate in 11 mm embryo^25^. In a 14 mm embryo, there are two separate compartments: a proximal group for GM, SM, PM and a deep group for the other posterior muscles of the leg. During development, the proximal group initially occupies a lateral position; from here, it gradually spreads to the medial side of the leg to attach to the tibia. Then, in the second half of the second month, the two separate heads of the GM develop, after which the PM separates from the lateral head^25^. Menton et al. suggest that the PM may also serve as a sensory organ^26^, to the extent that it can send afferent information about the position of the foot to the central nervous system. It is also possible that the GM performs this function in cases where the PM originates from the GM.

The second theory, based on the branching patterns of the nerves^27^, postulates that the nerve funiculus of the PM and the nerve elements of the SM share a close relationship with the nerve funiculi of the deep posterior crural muscles. In addition, the PM is usually innervated separately from the tibial nerve, whereas the GM and SM are usually innervated by a common trunk when they arise from the tibial nerve^27^. It has been suggested that the PM may develop independently and not from the GM^28^.

Despite its small size, PM has a high clinical significance. The annual incidence of PM injuries in athletes in the UK ranges from 3.9 to 9.3%, and it is possible that such injuries have been inadequately diagnosed^29^. Most plantaris ruptures occur at the junction between the PM belly and PM tendon^9^. The PM tendon is also commonly used as a donor for flexor tendon replacement in hand and ankle joints^8^. According to Yammine et al.^30^ PM tendon is a large-sized and consistent source of tendon graft. In most reconstruction, surgeons use Palmaris longus as a graft, however PM occur more frequently than Palmaris longus. Thus PM is more reliable for reconstruction or grafting. Furthermore, PM has longer tendon in comparison with Palmaris longus, what is crucial during surgeries. The type of the tendon may be confusing during harvesting procedures due to its complexity^7^. For this reason it is so important to know the type of the PM tendon insertion. Moreover, PM affects Achilles tendinopathy in more than half of cases^31^. Some studies even underline the correlation between the type of PM tendon and the Achilles midportion tendinopathy^31,32^. According to Alfredson and Spang^32^, in 41% of patients suffering from Achilles tendinopathy, PM tendon was located close to the medial side of the midportion of the Achilles tendon. Van Sterkenburg et al.^31^ noted that at the level of the Achilles midportion tendinopathy PM tendon and calcaneal tendon were located closely.

The classification of PM tendons used in the present study is based on the six-fold classification of Olewnik et al.^4,11^, with some modifications. The original classification was based on 50 lower limbs^4^ and 130 lower limbs^11^ from adult cadavers (Table 3). We used this classification because of its accuracy, diversity, and presence in other literature. The same classification was also used by Szaro et al. to classify the PM insertion in 72 lower limbs from human fetuses (Table 3)^33^. Whereas the most common type recorded by Olewnik et al. was type I (44%), type II was recorded by Szaro et al. (34.7%) and the present study (21.74%).

**Table 3:** Comparison of the insertion of the PM (Olewnik et al.^11^; Szaro et al.^33^).

	Olewnik et al.	Szaro et al.	Current study
Type I	44%	30.6%	9.78%
Type II	22.4%	34.7%	21.74%
Type III	6.9%	8.3%	2.17%
Type IV	3.4%	9.7%	20.65%
Type V	18.1%	4.2%	8.7%
Type VI	5.2%	0%	2.17%
Type VII	0%	0%	10.87%
Type VIII	0%	0%	2.17%

In the present study, two additional types were identified: Type VII and Type VIII. In type VII, the PM tendon runs anterior to the Achilles tendon and attaches to it. In one case, the PM tendon was 42.6 mm long before dividing into two bands: Band I (3.28 mm) and Band II (7.68 mm), both of which terminated in the Achilles tendon. Type VIII is characterized by a short tendon that attaches to the SM. The differences in the percentage of each kind of PM tendon is difficult to explain. Possibly it might be a result of developmental factors or be related to the group selection.

In addition to Szaro et al. two other studies have investigated PM in human fetuses^16,17^. Yildiza et al. examined 24 human fetuses and Desdicioglu et al. examined 51 human fetuses, and both studies mainly consider the morphometrics of the PM tendon. The mean lengths of the PM tendon in the second and third trimesters are compared in Table 4. The mean length of the diameter of PM tendon in 2nd trimester was the shortest in Yildiza et al. study, whereas in current study mean length in 2nd was the longest^16^. In 3rd mean length of PM tendon was the longest in Yildiza et al. study, whereas in Desdicioglu et al. study was the shortest^17^. Length of PM tendon was similar in ours and Desdicioglu et al.’s reports, however there was a difference between these two studies and Yildiz et al.’s study: this might be a result of the difference in number of the investigated fetuses in those studies because we and Desdicioglu et al. studied more individuals than Yildiza et al. Neither Yildiza et al. nor Desdicioglu et al. identified all insertion types as reported by Olewnik et al. Nevertheless, Yildiza et al. reported a case of a very short tendon that inserted at the fibula head: the tendon was 2.11 mm long and 0.2 mm wide. In another case, the PM tendon terminated in the Achilles tendon^16^. Desdicioglu et al. reported that in 19.61% of cases, the PM ended as a fusion with the calcaneal tendon^17^. In present study, this type of insertion, classified as type VII, was observed in 10.87% of cases.

**Table 4:** Comparison of PM tendon length as a function of trimester of gestation.

	Desdicioglu et al.	Yildiza et al.	Current study
2nd trimester	46.6 mm	36.35 mm	42.46 mm
3rd trimester	49.92 mm	65.39 mm	54.29 mm

A significant study of the course and insertion of the PM tendon, performed on 750 lower limbs, was presented by Daseler et al.^24^. The authors distinguished four types of insertion: Type I, in which the tendon terminates as a short flare in the medial extremity of the superior tuberosity; Type II, in which the tendon insertion is at the calcaneum anterior to the adjacent border of the Achilles tendon; Type III, in which the insertion is medial to the terminal portion of the Achilles tendon and calcaneum; Type IV, in which the tendon inserts into the medial aspect of the Achilles tendon^24^.

Dos Santos et al. proposed a threefold classification of the PM tendon^34^, two types based on medial and anteromedial insertions to the calcaneum and another type inserting into the Achilles tendon proximal to the calcaneum^34^, this types are similar to our types I, II and III. Nayak et al. describe insertion of the PM into the Achilles tendon, retinaculum flexorum, and independently into the calcaneum, this types are similar to our types I, II, III and VI. Van Sterkenburg et al. define nine types of insertion based on tendon morphology and insertion type: anterior to the calcaneus, medial to the calcaneus, medial and fan-shaped to the calcaneus, medial to the Achilles tendon, medial with thin slips to the calcaneus, anteromedial to the calcaneus, anteromedial and fan-shaped to the calcaneus, or as deep fascia^31^. Finally, Gonera et al.^8^ and Kurtys et al.^35^ describe single case reports in which the distal attachment of the PM was present with multiple bands and different insertion points, what was similar to our type VII, in one case, the PM tendon was divided into two ligaments before attachment to the Achilles tendon.

This study has some limitations. Because PM insertion is so variable, the proposed classification is heterogeneous and depends on several morphological details, such as the presence of an accessory division or the type of insertion. In addition, the optimal sample size was not calculated. Nevertheless, the present study is one of the largest to date and the first such study in human fetuses. Our systematic classification can be used to improve the outcomes of future leg and foot interventions and also to study the morphological variations occurring in the human body.

## Conclusions

We propose an eight-fold classification of PM insertion in fetuses. Seven of the included types were previously described in adults; however, one additional type was added: Type VIII, in which the tendon is short and attaches to the SM. Leg length, length of tendon, extension point (ExP) from the calcaneus, and ExP thickness differed significantly among types of PM insertion.

## Data Availability

Please contact authors for data requests (Anna Waśniewska-Włodarczyk email address: anna.wasniewska@umed.lodz.pl).

## Abbreviations

SM: Soleus muscle
PM: Plantaris muscle
GM: Gastrocnemius muscle
ExP: Extension point
AT: Achilles tendon
SD: Standard deviation
